# Identification of a novel envelope protein encoded by *ORF 136* from Cyprinid herpesvirus 3

**DOI:** 10.1007/s00705-017-3528-5

**Published:** 2017-08-16

**Authors:** Shucheng Zheng, Yingying Li, Qing Wang, Jiexing Wu, Yingying Wang, Weiwei Zeng, Sven M. Bergmann, Yan Ren, Cunbin Shi

**Affiliations:** 1 Key Laboratory of Fishery Drug Development of Ministry of Agriculture, Key Laboratory of Aquatic Animal Immune Technology of Guangdong Province, Pearl River Fisheries Research Institute, Chinese Academy of Fishery Sciences, Liwan District, Guangzhou, 510380 People’s Republic of China; 2 College of Fisheries and Life Science, Shanghai Ocean University, Shanghai, People’s Republic of China; 3 German Reference Laboratory for KHVD, Institute of Infectology, Friedrich-Loffler-Institut (FLI), Federal Research Institute for Animal Health, Südufer 10, 17493 Greifswald-Insel Riems, Germany

## Abstract

Cyprinid herpesvirus 3 (CyHV-3) is the pathogenic agent of koi herpesvirus disease (KHVD) afflicting common carp and koi (*Cyprinus carpio* L.) populations globally. As described previously, proteomic analyses of purified CyHV-3 particles have shown that at least 46 structural proteins are incorporated into CyHV-3 virions; among these *ORF136* may encode a putative envelope protein. In this study, Western blotting analysis showed that a specific band with the predicted molecular weight of 17 kDa was detected both in purified virions and envelope components using a rabbit anti-ORF136 polyclonal antibody. Indirect immunofluorescence assay with confocal laser scanning microscopy indicated that the ORF136 protein was distributed in the cytoplasm of CCB cells infected with CyHV-3 and transfected with a pVAX1-ORF136 plasmid. Furthermore, immunogold electron microscopy confirmed that ORF136 protein localized to the CyHV-3 envelope.

Koi herpesvirus disease (KHVD), caused by Cyprinid herpesvirus 3 or Koi herpesvirus, results in considerable financial losses in common carp and koi (*Cyprinus carpio* L.) farming industries in Asia [4, 8, 14], Western Europe [2, 7], the United States [8] and other countries or regions. Recently, KHVD has also occurred in Vietnam [9] and Iran [11] where it has never previously been detected, suggesting that this destructive disease remains a tremendous threat to carp or koi populations worldwide.

Taxonomically, CyHV-3 is classified as a member of the genus *Cyprinivirus* within the family *Alloherpesviridae* in the order *Herpesvirales* that also includes Cyprinid herpesvirus 1 (CyHV-1), Cyprinid herpesvirus 2 (CyHV-2), and Anguillid herpesvirus 1 (AnHV-1) [3]. More knowledge associated with diagnosis and detection has been developed since its first identification in the United States and Israel in 1998 [8], whereas fundamental research such as the study of the biological function of structural proteins incorporated into CyHV-3 virions, host-virus interactions and mechanisms of pathogenesis are still largely limited. CyHV-3 is an enveloped virus with an approximately 295 kbp double-stranded DNA encoding 156 open reading frames (ORF) [1]. Proteomic analyses of purified CyHV-3 virions have shown that at least 46 proteins including 2 tegument, 3 capsid, 16 envelope and 25 unknown proteins are incorporated into mature CyHV-3 virions [10, 18]. Although a number of predicted structural proteins are present in CyHV-3, most of them have not been identified with regard to their localization and biological function; knowledge that would improve our understanding of key aspects of the viral life cycle such as virus entry, assembly and replication. Up to now, a number of structural proteins associated with CyHV-3 have been identified as envelope proteins (ORF81 [12], ORF83 [18], and ORF149 [5]) and capsid proteins (ORF92 [18]), while other putative type I membrane glycoproteins encoded by *ORF25*, *ORF65*, *ORF148* and *ORF149* have been considered as immune-relevant membrane proteins [5]. However, one of the putative membrane glycoproteins, the ORF136 protein could not be recognized by sera from naturally or experimentally CyHV-3-infected koi using indirect immunofluorescence assays after transient expression in Rabbit kidney (RK13) cells, which appeared to show its lack of immunogenicity or low abundance in CyHV-3-infected cells [5].

Despite this, one of our previous studies based on proteomic analysis of separated envelope fractions, showed ORF136 was one of the most abundant proteins localized in the envelope (unpublished). In addition, ORF136 has also been characterized in CyHV-3 particles by mass spectrometry analyses with higher emPAI values, as reports have described previously. Therefore, we speculate that ORF136 may be an essential structural component in CyHV-3 virions. In this study, ORF136 was further characterized using a series of methods to determine its localization using a rabbit anti-ORF136 polyclonal antibody prepared previously. This research may lay the foundation for future functional studies of ORF136.

Common carp brain cell line (CCB) were cultured in Dulbecco’s modified Eagle’s medium (DMEM) supplemented with 10% fetal bovine serum (FBS) and incubated at 27 °C with 5% CO_2_. The rabbit anti-ORF136 polyclonal antibody developed previously in our laboratory was used in this study. The CyHV-3 isolate used in this experiment was isolated in April 2013 from moribund koi at a farm in Guangzhou, China, and named GZ1301. CCB cells were infected with CyHV-3 as described previously with minor modifications [16]. Adsorption of CyHV-3 was performed for 1 h at 27 °C. The virus was harvested for purification after the appearance of acute and apparent cytopathic effect was observed. The supernatants and cultures were exposed to repeated freeze-thaw cycles at −80 °C and then centrifuged at 10000 rpm/min for 30 min at 10 °C to completely remove the cell debris. The supernatants were centrifuged by ultracentrifugation at 32000 rpm/min for 90 min at 10 °C in a type 70 Ti rotor (Beckman coulter). The resultant pellets were resuspended in TN buffer (10 mM Tris, 10 mM NaCl, pH 7.4.) and layered onto 20-66% discontinuous sucrose gradients for ultracentrifugation at 25000 rpm/min for 1 h at 10 °C in a SW 41Ti rotor (Beckman coulter). The vast majority of virion bands localized around the 50-66% sucrose gradient were collected, rinsed with TN buffer and centrifuged at 25000 rpm/min for 30 min at 10 °C. Subsequently, the pellets were resuspended in TN buffer and were observed with transmission electron microscope with negatively stained 3% phosphotungstic acid (pH 7.2~7.4) for 1 min.

Extraction of envelope fractions from purified virions was carried out using 1% TritonX-100 dissolved in TN buffer. The virion mixture was incubated at room temperature for 30 min followed by centrifugation at 18000×g for 2 h at 4 °C. The supernatants were collected completely and pellets were resuspended in TN buffer. Both of the separated samples were stored at −20 °C for further analysis.

Purified virions, envelope fractions and pellet fractions were treated with 5× Sample Buffer (60 mM Tris-Cl pH 6.8, 2% SDS, 10% glycerol, 5% β-mercaptoethanol, 0.01% bromophenol blue) for 5 min in boiling water and were then analyzed on Novex NuPAGE Bis-Tris Pre-Cast polyacrylamide gels (10%) (Invitrogen) followed by staining with coomassie brilliant blue.

The samples were separated by sodium dodecyl sulfate polyacrylamide gel electrophoresis (SDS-PAGE) and transferred to nitrocellulose membrane (Merck Millipore, Ireland). Subsequently, the membrane was blocked for 1 h at room temperature with 5% skimmed milk dissolved in phosphate buffer solution (PBS) supplemented with 0.05% Tween-100 (PBST). The membrane was incubated with the rabbit polyclonal antibody against ORF136 recombinant proteins at a dilution of 1:1000 for 1 h at room temperature. After washing with 0.05% PBST three times, the membrane was incubated with horseradish-peroxidase (HRP) conjugated goat anti-rabbit IgG (Boster, China) at a dilution of 1:10000 for 1 h at room temperature. After washing with 0.05% PBST three times, the membrane was stained with an enhanced chemiluminescent commercial kit, according to the manufacturer’s instructions (Thermo scientific).

For construction of the recombinant eukaryotic expression vector, the complete *ORF136* was amplified from the CyHV-3 genome by PCR. Amplifying primers (Forward: 5′-CTCTAGACTCGAGATGAAGGCCTCTAAACT-3′, Reverse: 5′-GTGGTGGAATTCTTAGATTTTTCTAAAGTG-3′; restriction enzymes X*ho*I and E*co*RI sites are underlined, respectively) were designed based on published CyHV-3 genomic sequence. After digestion, the PCR products were inserted into eukaryotic vector pVAX1 digested with the corresponding restriction enzymes. The recombinant eukaryotic vector sequence was determined by sequencing. Finally, CCB cells were transfected with recombinant eukaryotic plasmid pVAX1-ORF136 according to the manufacturer’s instructions for the transfection reagent (Takara).

CCB cells infected with CyHV-3 and transfected with recombinant eukaryotic plasmid pVAX1-ORF136 were examined by indirect immunofluorescence assay. Culture medium was removed from cover slides followed by washing with PBS to clean up residual cell debris. Cells were fixed with ice cold methanol-acetone mixture (1:1) for 10 min at −20 °C and completely dried at room temperature. Subsequently, the cells were blocked for 1 h at room temperature with 10% skimmed milk dissolved in PBS. After washing with 0.05% PBST, the cells were incubated with rabbit anti-ORF136 polyclonal antibody at a dilution of 1:100 with PBS for 1 h at room temperature. The cells were rinsed with 0.05% PBST three times and incubated with Alexa Fluor488 conjugated donkey anti rabbit antibody (Invitrogen, USA) at a dilution of 1:100 with PBS for 1 h at room temperature. After a final wash with 0.05% PBST three times, the cell nuclei were stained with 4′,6′-diamidino-2′-phenylindole (DAPI) (Beyotime Biotechnology, China) for 10 min at room temperature and were analyzed using confocal laser scanning microscopy (Carl Zeiss, Germany).

Purified CyHV-3 virions were adsorbed onto formvar/carbon film coated nickel grids (200-mesh) (Zhongjingkeyi, China) for 15 min at room temperature. Residual virions were removed from grids and air dried at room temperature. The nickel grids were blocked with 3% BSA for 1 h at 37 °C and then rinsed with PBS. The rabbit anti-ORF136 polyclonal antibody, at a dilution of 1:100 and the gold-labeled anti-rabbit goat IgG (Sigma-Aldrich) at a dilution of 1:100, were incubated at 37 °C for 1 h, respectively. Negative rabbit serum was used as control in the same way. PBS was used as the abluent for each step after incubation with antibody. Finally, grids were negatively stained with 3% phosphotungstic acid (pH 7.2~7.4) for 1 min and observed with transmission electron microscope.

As results of the SDS-PAGE showed, notable differences were observed between the envelope fraction and purified virions (Fig. 1(a)). One of the most evident marker proteins, the major capsid protein encoded by *ORF92* was, as expected, present in purified virions but not present in envelope fractions. Immune electron microscopy analysis further indicated that pellets had typical naked nuclocapsids (results not shown).

**Fig. 1:**
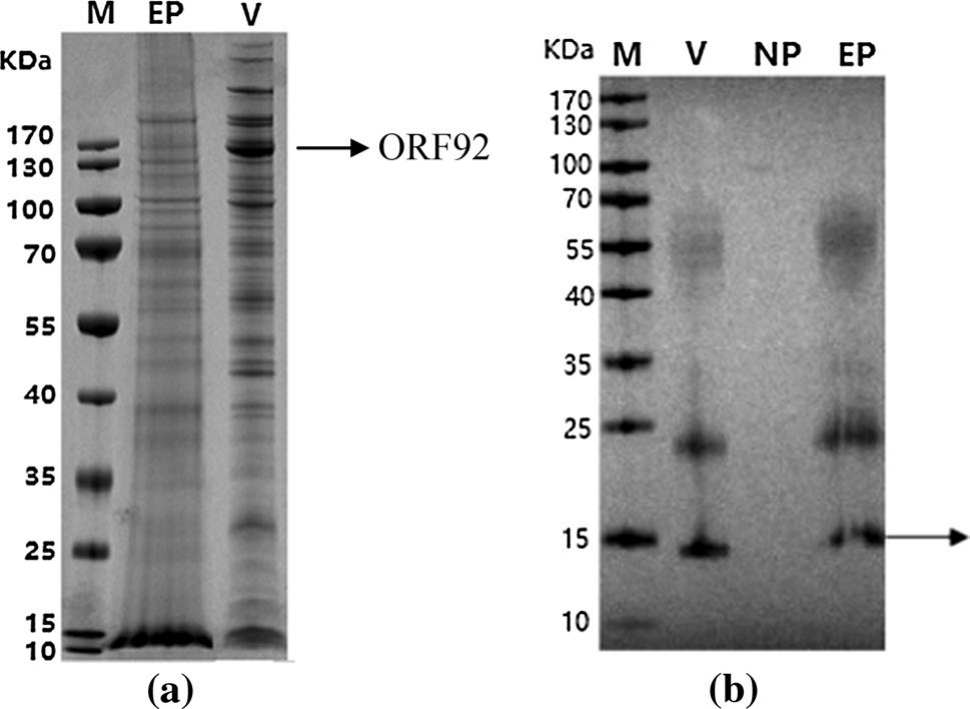
SDS-PAGE of viral components and Western blotting analysis. Separation by 10% SDS-PAGE gel with visualization by coomassie brilliant blue (a) or western blotting analysis of CyHV-3 ORF136 using the rabbit anti-ORF136 polyclonal antibody (b). The arrow in Fig. 1 (b) indicates the specific band of ORF136. M: Marker; V: purified CyHV-3; EP: envelope; NP: nucleocapsid

Proteins from CyHV-3 virions, envelope and nuclocapsid were separated by 10% SDS-PAGE followed by western blotting analysis. After western blotting analysis, a specific band around 15 kDa was detected both in purified virions and the envelope compartment after application of the rabbit anti-ORF136 polyclonal antibody, which closely matched the expected molecular mass of ORF136 – 17 kDa (Fig. 1(b)), indicating that the ORF136 protein was distributed in the viral envelope. Of note, specific bands were not detected in purified virions or envelope using pre-immune sera from rabbit (data not shown).

To further clarify the subcellular localization of ORF136, indirect immunofluorescence assays with confocal laser scanning microscopy were performed. As results show in Fig. 2, green fluorescence was distributed in the cytoplasm of CCB cells both infected with CyHV-3 (Fig. 2(a)) and transfected with pVAX1-ORF136 plasmid (Fig. 2(c)), while no green fluorescence signal was detected in uninfected cells (Fig. 2(b)) or the control plasmid transfected cells (Fig. 2(d)), revealing the cytoplasmic localization of the ORF136 protein. As a negative control, a pre-immune serum from rabbit was used as a primary antibody. No specific signal was detected in these corresponding samples (data not shown).

**Fig. 2:**
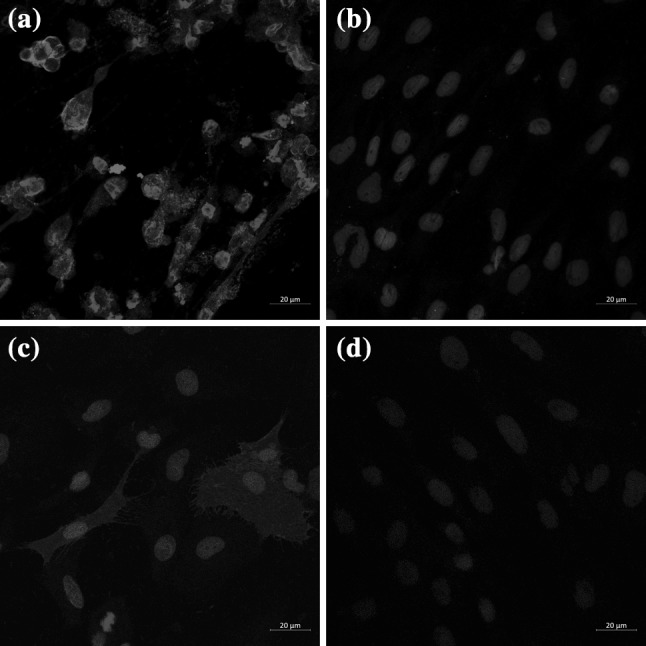
Indirect immunofluorescence assay with confocal laser scan microscopy. CCB cells were infected (a) or uninfected (b) with CyHV-3, or were transfected with pVAX1-ORF136 plasmid (c) and control plasmid pVAX1 (d). The specific signals were detected with Alexa Fluor 488-conjugated secondary antibodies (green) and nuclei were stained with DAPI (blue). Bars: 20 um (color figure online)

Localization of ORF136 by transmission electron microscopy was carried out using purified CyHV-3 virions. The gold particles could be found on the envelope of purified CyHV-3 virions following incubation with the specific polyclonal antibody against ORF136 (Fig. 3 (a)), whereas no gold particles were observed on the viral envelope using negative rabbit serum as a primary antibody (Fig. 3 (b)). Taken together, this demonstrates that ORF136 was identified as an envelope protein of CyHV-3.

**Fig. 3:**
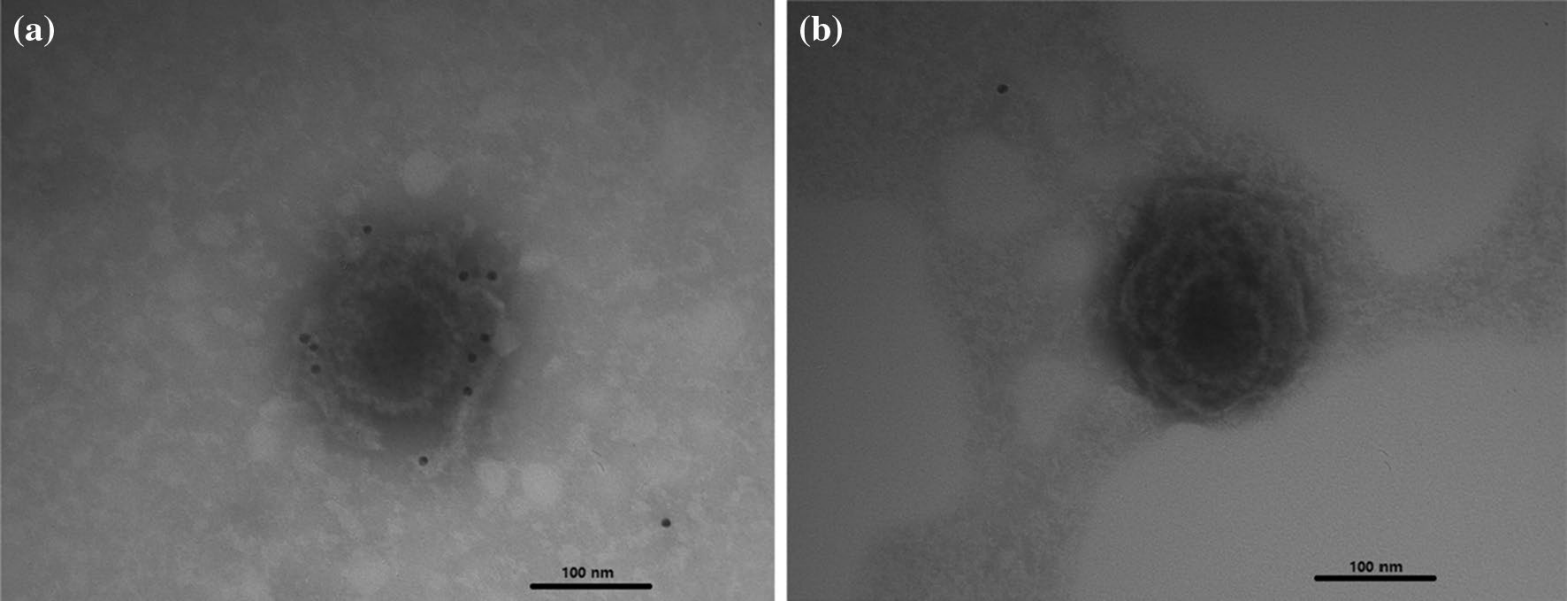
Immunogold electron microscopy of CyHV-3 particles. Purified CyHV-3 virions were incubated with the ORF136 polyclonal antibody (a) or negative rabbit serum (b) and gold particles conjugated to a secondary antibody. Bars: 100 nm

Characterization of structural proteins may increase our understanding of the relationship between host and virus, helping to elucidate mechanisms of pathogenesis and develop effective prevention and control strategies against the disease. However, few of the predicted structural proteins of CyHV-3 have been characterized, with respect to localization, function and protein-protein interactions. In the present study, ORF136 unambiguously localized as a novel envelope protein of CyHV-3. Bioinformatics software predicted that the ORF136 protein has 5 N-glycosylation sites, 3 N-myristoylation sites and one microbodies C-terminal targeting signal. More functional characterization should be performed to confirm whether the ORF136 protein undergoes protein processing and post-translation modifications. In addition, it is well known that envelope proteins are involved in membrane fusion between the viral envelope and host cellular membrane for entry of herpesvirus, for instance herpes simplex virus-1 (HSV-1) [6], human cytomegalovirus (HCMV) [13] and Epstein-Barr virus (EBV) [15]. However, little research has been performed concerning the molecular mechanisms of fish alloherpesviruses, due to the scarcity of knowledge relating to their envelope proteins. Our study showed that the nonionic surfactant TritonX-100 could effectively separate viral envelope in line with other previous reports [17, 19], which may provide essential technical understanding for the identification and characterization of other putative envelope proteins.

The recombinant ORF136 protein expressed in prokaryotic cells as well as the recombinant eukaryotic plasmid pVAX1-ORF136 could be used as effective targets for preparing a subunit vaccine or DNA vaccine, respectively. Moreover, the polyclonal antibody specific to ORF136 protein might be used as a diagnostic antibody for development of ELISA to capture antigen.
